# Dilated versus non‐dilated left ventricular cardiomyopathy: Same same but different?

**DOI:** 10.1002/ehf2.14923

**Published:** 2024-07-09

**Authors:** Michał Tkaczyszyn

**Affiliations:** ^1^ Institute of Heart Diseases Wroclaw Medical University Wroclaw Poland; ^2^ Institute of Heart Diseases University Hospital Wroclaw Poland

Non‐dilated left ventricular cardiomyopathy (NDLVC) [previous nomenclature proposed by the European Society of Cardiology Working Group on Myocardial and Pericardial Diseases was hypokinetic non‐dilated cardiomyopathy (HNDC)] constitutes a relatively new clinical entity defined and introduced due to the fact that many patients suspected of still subclinical or potentially developing dilated cardiomyopathy (DCM) (e.g., relatives of patients with DCM harbouring pathogenic/likely pathogenic genetic variant) do not meet all diagnostic criteria (e.g., the left ventricle [LV] has only subtle systolic dysfunction and is not enlarged—the so‐called intermediate phenotypes) but definitely require vigilant supervision/follow‐up or even already specific treatment to be initiated.^1^, ^2^ There was indeed an important practical need to group a broad range of patients with variously distributed LV scarring, isolated global impairment of LV systolic function (frequently only slight), or LV dilatation with preserved left ventricular ejection fraction [LVEF], in one diagnosis, intuitively considered as a form preceding the development of overt, symptomatic DCM.^1^, ^2^, ^3^ However, should NDLVC really be considered a ‘milder’ clinical entity?

In the recent research published in ESC Heart Failure, Eda and et al.^4^ present important additional data on this topic. The authors compared the clinical characteristics and major cardiac events on a retrospective basis in 283 DCM patients (LVEF<50% + left ventricular end‐diastolic diameter [LVEDD] index  > 31 and 34 mm/m^2^ in females and males, respectively) and 80 patients with NDLVC (LVEF<50% + LVEDD index below above cut‐offs) who were followed‐up for a median of 69 months, and demonstrated that despite obvious differences in echocardiography and cardiac magnetic resonance (CMR) imaging, patients with NDLVC do not have a better outcomes than patients with DCM in terms of the composite primary endpoint comprising sudden cardiac death and hospitalization due to heart failure.^4^ It needs to be acknowledged that all enrolled patients were newly diagnosed (<6 months) with LV cardiomyopathy. This is of particular importance because in cardiomyopathy cohorts without the information when the diagnosis was firstly established, it is possible that long‐term cardioprotective and anti‐remodelling treatment (initiated, e.g., years before an index evaluation) could have led to a reduction in LV dimensions and an improvement regarding its initial systolic dysfunction. Therefore, in the group of patients considered as NDLVC for the cross‐sectional analysis purposes, there may be actually many patients who met initially DCM criteria but at the time of the evaluation present with improved LV morphology and/or contractility. Importantly, there was also a clear trend towards worse long‐term prognosis in patients with NDLVC who developed ventricular dilatation during follow‐up (interestingly, in most of these patients overt dilatation occurred in a relatively short period of time, i.e., 6–12 months).^4^ Data presented in cited research have two important clinical implications. Firstly, it may not be entirely appropriate to consider LVNDC as a more benign disease [even despite mild symptomatology—almost 50% of aforementioned patients were assigned New York Heart Association (NYHA) class I] compared with overt, symptomatic DCM (2/3 of such subjects in cited research were at least NYHA II). Secondly, adverse left ventricular remodelling despite administered cardioprotective treatment (both groups were treated comparably regarding % of major cardioprotective drugs) can predict increased risk of cardiac events.

Although the sub‐group of NDLVC (previously HNDC) was separated from DCM a few years ago, we still have very little clinical data on this subject and increased clinical interest should be expected after clearly highlighting these patients in the recent European cardiomyopathy guidelines.^2^ The limited available data on the prognosis of NDLVC versus DCM are, however, inconclusive. In one of a few research work referring to this clinical problem Dziewięcka et al.^5^ retrospectively compared clinical characteristics and outcomes in ‘classical’ DCM patients and patients with NDLVC (classification based on LVEDD >52 mm and 58 mm in women and men, respectively) who were followed‐up for a period of 47 ± 31 months. Although DCM patients presented with more ventricular arrhythmia burden, greater neurohormonal activation and were treated with higher daily doses of diuretics, in terms of a composite endpoint comprising all‐cause death, heart transplantation or left ventricular assist device implantation, there were no significant differences between analysed two groups.^5^ In another recent study Castrichini et al.^6^ comprehensively analysed retrospective clinical, CMR and genetics data of almost 500 patients with DCM and NDLVC from 4 centers from the Italy and the United States.^6^ NDLVC was diagnosed literally according to new guidelines^2^ criteria, and LV dilatation was defined as left ventricular end‐diastolic volume index >96 mL/m^2^ for female and >105 mL/m^2^ for male patients. Intriguingly, although NDLVC patients had greater prevalence of pathogenic or likely pathogenic variants of arrhythmogenic genes and more frequently reported sudden cardiac death (SCD) episodes in family history, their prognosis in terms of SCD or major ventricular arrhythmias was better than classical DCM phenotype.^6^ However, the above‐cited data are retrospective and there is still a large unmet need for prospective follow‐up of patients with NDLVC versus DCM to better understand the clinical trajectories and common elements of both LV disease phenotypes, especially at oligosymptomatic stage, when the critical element of the assessment is SCD risk stratification.

Indeed, another important clinical challenge should be addressed here, namely the comprehensive assessment of prognosis (especially major arrhythmic events/SCD, but also cardiac decompensation, clinical deterioration leading to advanced heart failure with the need for mechanical circulatory support or heart transplantation) in patients with non‐ischemic left ventricular cardiomyopathy (both DCM and NDLVC)^7^, ^8^, ^9^ (*Figure* *1*). While certain recommendations and diagnostic‐therapeutic schemes regarding DCM population have been developed and published,^10^ there are no randomized clinical trials data specifically on NDLVC patients. Moreover, there is increasing evidence that precise risk stratification of life‐threatening arrhythmic events in non‐ischaemic LV cardiomyopathy is much more difficult than previously thought and this risk is most likely a function of overlapping patient genetics, the characteristics and distribution of myocardial damage and ventricular arrhythmia burden at the time of assessment, but also possibly the multidimensional effects of the use (or not) of contemporary evidence‐based anti‐remodelling and cardioprotective therapies (e.g., heart failure foundational drugs and cardiac resynchronization therapy).^11^, ^12^, ^13^, ^14^, ^15^ Undoubtedly, the prognostication in patients with left ventricular cardiomyopathies will evolve dynamically as new empirical data are obtained due to the increasing availability of high‐quality multimodality imaging and genetic profiling.^16^, ^17^, ^18^

**Figure 1:**
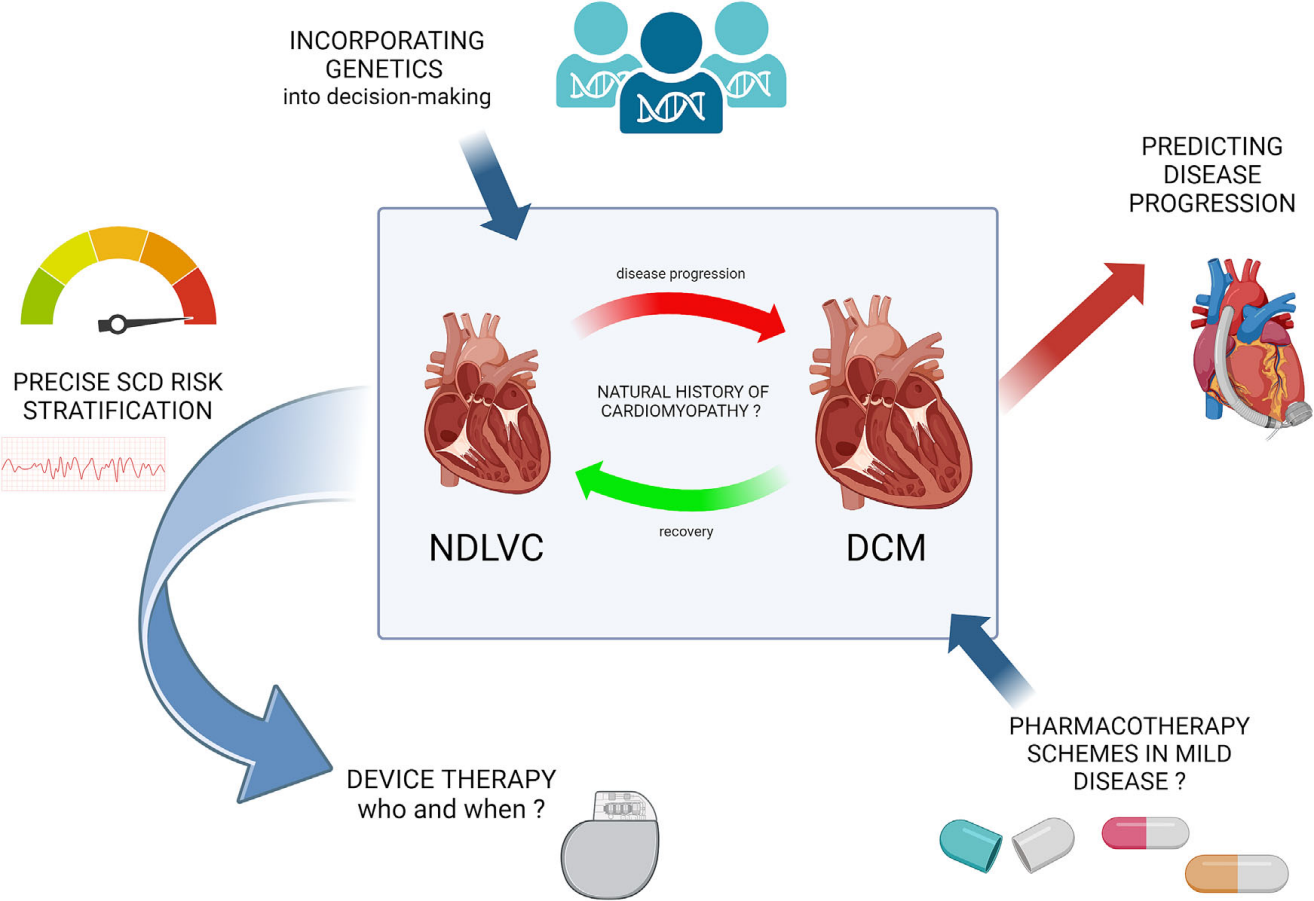
Major clinical challenges regarding the transition or not from non‐dilated left ventricular cardiomyopathy (NDLVC) to overt dilated cardiomyopathy (DCM). SCD, sudden cardiac death. Created with BioRender.com.

## Conflict of interest

Dr Tkaczyszyn reports personal fees from V‐Wave Ltd., Eidos Therapeutics, Cytokinetics, Impulse Dynamics, Alnylam Pharmaceuticals, and Takeda, outside the submitted work.
